# Simulation of thermal hazards risk in octogen based on non-isothermal DSC data

**DOI:** 10.1038/s41598-023-48372-2

**Published:** 2023-12-01

**Authors:** Zhi Wang, Shaohua Jin, Lijie Li, Hui Chao, Shichuan Qian, Xinping Zhao, Xin Sheng, Zhiyan Lu, Guanghui Gu, Shusen Chen, Kun Chen

**Affiliations:** 1 School of Materials Science and Engineering, Beijing Institute of Technology, Beijing, China; 2 Gansu Yinguang Chemical, Industry Group Co., Ltd, Baiyin, China

**Keywords:** Energy, Materials for energy and catalysis

## Abstract

To evaluate the possible thermal risks associated with the storage of octogen (HMX), non-isothermal differential scanning calorimetry (DSC) experiments were conducted in order to ascertain the kinetic model and parameters governing its thermal decomposition. DSC measurements indicate that HMX underwent a crystal transformation prior to thermal decomposition. A kinetic model for the autocatalytic thermal decomposition process was developed through the analysis of its primary exothermic peaks. Subsequently, numerical simulations were performed using the aforementioned kinetic model to assess the potential thermal explosion hazard of HMX under two distinct storage conditions. The comparison was made between the models of HMX autocatalytic decomposition temperature and thermal explosion critical temperature under two distinct storage conditions. The prediction of the influence of ambient temperature on the critical temperature of thermal explosion is conducted simultaneously. Finally, the thermal hazard parameters of HMX under different package quality are given.

## Introduction

The thermal runaway reaction during the fabrication, storage, transportation, and destruction of hazardous chemicals has been known to cause numerous fatalities and injuries. In 2015, a tragic incident took place in Tianjin, China, where a hazardous goods warehouse experienced multiple explosions, resulting in the loss of 173 lives. In the year 2020, a tragic incident occurred in the port vicinity of Beirut, Lebanon, resulting in the loss of over 200 lives. These incidents serve as a reminder of the importance of properly storing hazardous substances.

Octogen (HMX) is a byproduct that was isolated from the process of preparing hexogen (RDX) from acetic anhydride, as documented by Bachmann W. E. et al. in 1941^1^. The density of HMX is 1.903 g/cm^3^, the detonation velocity is 9110 m/s, and the 5 s delay explosion is 327 °C^2^. According to the existing literature, the activation energy (E_a_) of the thermal decomposition of HMX has been experimentally determined to range from 42 to 1070 kJ/mol, and the decomposition temperature ranged between 268 and 314 °C^3–5^. Due to its favorable chemical stability and exceptional thermal stability, HMX finds extensive application in high-performance explosives, solid propellants, and gun propellant. As one of the most widely utilized energetic materials, ensuring the storage safety of HMX^6^ is of paramount importance. Starting from the discovery of HMX, efforts have been devoted to exploring the kinetics and mechanism of its thermal decomposition^7–11^, which are crucial for understanding the stability of HMX, its combustion process, and evaluating the safety of its storage condition.

Thermal analysis techniques such as DTA, TG, ARC, and DSC have been primarily used in studying explosive decomposition kinetics under isothermal and non-isothermal conditions^3,12–15^. Due to variations in testing and calculation methods, the reported activation energy values in the literature exhibit a wide range^5^. Zhao et al.^16^ utilized a nonlinear optimization method to analyze non-isothermal DSC data and investigated the thermal decomposition kinetic model of CL-20/HMX co-crystal. The critical temperature of the 1000th-second explosion was determined and the impact of different packaging materials on the self-accelerating decomposition temperature (SADT) was simulated based on the thermal decomposition kinetic model. In addition, Zhang et al.^17^ developed a thermal decomposition kinetics model for *N*-nitrodihydroxyethyl dinitrate (DINA) by combining the results of DSC and ARC tests, and a numerical simulation based on kinetics was conducted to evaluate the potential risk of thermal explosion for DINA under various conditions. However, there is currently no existing report on the safety assessment of HMX storage using kinetic models of thermal decomposition. The estimation of safety parameters through the utilization of thermal decomposition kinetics and thermal explosion models can offer valuable assistance in the prevention of accidents and the management of risks associated with the storage of HMX.

Herein, a decomposition kinetic model of HMX was obtained by performing dynamic DSC tests to predict the temperature-time profiles under different storage conditions. Due to the significant variability in activation energy and decomposition temperature reported in the literature, it is necessary to conduct DSC tests in order to obtain the thermal decomposition curve and determine the main safety parameters. The temperature-time profiles of a specific package were simulated using the Thermal Safety Software (TSS) to predict the effects of different storage conditions, based on the kinetic model and heat transfer conditions^18^. The kinetic model and safety parameters of HMX were utilized as a benchmark to further assess the potential risk of thermal damage in the event of an explosion during the transportation and storage of HMX.

## Experiment and method

### Sample

Industrial HMX was acquired in the form of a white powder and subsequently purified through recrystallization utilizing acetone.

### Differential scanning calorimetry (DSC) experiment

The differential scanning calorimeter (DSC) measurement was conducted on NETZSCH. In order to mitigate the potential damage to the apparatus caused by the pyrotechnic samples and to ensure the comparability of experimental data, all specimen amounts used in the DSC method tests were approximately 0.7 mg. The experiments were conducted under a continuous flow of nitrogen gas at a rate of 20 mL/min. The tests were carried out using an open-ended aluminum crucible, and the heating rates employed were 4, 6, 8, and 10 K/min, respectively.

### Establishment of thermal decomposition kinetics model

Choosing the appropriate model type plays an important role in constructing the correct kinetic model. It has been shown that the proper selection of the model type guarantees the reliability of the results. By conducting a comparison between the N-order reaction model and the autocatalytic reaction model, it has been determined that the autocatalytic reaction model provides a more comprehensive and precise representation of the thermal decomposition process of HMX. Therefore, the autocatalytic decomposition model is employed for the purpose of fitting. The experiment yielded limited information on the decomposition process, resulting in only a general understanding of the overall changes in the reaction. However, the specific changes in composition could not be determined due to the lack of detailed information. Therefore, it is necessary to employ a simplified kinetic model to describe the decomposition process. The formal reaction model, as proposed by Kossoy and co-researchers^19–22^, is considered the most appropriate method for this study. Although this model lacks the ability to provide a detailed mechanism, it is capable of accurately describing the primary characteristics of the reaction. The autocatalytic reaction is most simply described as:

$$A\xrightarrow{{k_{1} }}C + D$$

$$A{\text{ }} + {\text{ }}C\xrightarrow{{k_{1} }}{\text{ }}C{\text{ }} + {\text{ }}E$$

where substance C is the catalyst for reactant A. The total reaction rate is given by the following equation:

1 $$- r_{A} = k_{1} c_{A}^{{n_{1} }} + k_{2} c_{A}^{{n_{2} }} c_{c}^{{n_{2} }}$$

where, ${r}_{A}$ is the decomposition rate of A; ${k}_{1}$ is the reaction rate constant of elicitation phase; ${k}_{2}$ is the reaction rate constant of autocatalytic phase; ${c}_{A}$ and ${c}_{C}$ are the instantaneous concentration of A and C, respectively; assuming that the number of reaction orders of the reactant A is ${n}_{1}$ in both the reaction elicitation phase and the autocatalytic phase; ${n}_{2}$ is the number of reaction orders of substance C in the autocatalytic phase. Equation (1) can be expressed as^19^:

2 $$\frac{{{\text{d}}\alpha }}{{{\text{d}}t}} = A_{2} {\text{e}}^{{ - \frac{{E_{2} }}{RT}}} \left( {1 - \alpha } \right)^{{n_{1} }} \left[ {(A_{1} {\text{e}}^{{ - \frac{{E_{1} }}{RT}}} \begin{array}{*{20}c} {/A_{2} {\text{e}}^{{ - \frac{{E_{2} }}{RT}}} } \\ \end{array} ) + \alpha^{{n_{2} }} } \right]$$

*t* is the time (s), ${A}_{1}$, ${A}_{2}$ are the pre-exponential factor at reaction conversion α for the elicitation phase and autocatalytic phase, respectively. ${E}_{1}$, ${E}_{2}$ are the apparent activation energies of the elicitation and autocatalytic phases, respectively.

Such that $z_{0} = A_{1} /A_{2}$, ${z}_{0}$ stands for ratio of pre-exponential factors. When ${z}_{0}{e}^{-\frac{{E}_{z}}{RT}}$ increases, the autocatalytic property becomes more intense. ${E}_{z}={E}_{1}-{E}_{2}$, assuming $A={A}_{2}$, $E={E}_{2}$. Equation (2) can be changed to:

3 $$\frac{{{\text{d}}\alpha }}{{{\text{d}}t}} = A{\text{e}}^{{ - \frac{E}{RT}}} \left( {1 - \alpha } \right)^{{n_{1} }} \left[ {z_{0} {\text{e}}^{{ - \frac{{E_{z} }}{RT}}} + \alpha^{{n_{2} }} } \right]$$

After data processing, they are imported into Fork software for calculation and optimization of kinetic parameters^23^. This model operates under the assumption that the conversion rate is a state variable within the reaction system. In the thermal explosion simulation, it is assumed that at initial instant temperature distribution within every zone and conversion distribution within every active zone are uniform, and all the conversions are equal to zero. These models fail to consider the alteration in the composition of the reaction mixture and the resulting modification in the physical properties of the system. The created kinetics is valid only for the mixture composition investigated.

## Results and discussion

### Thermal analysis by dynamic DSC

**Figure 1:**
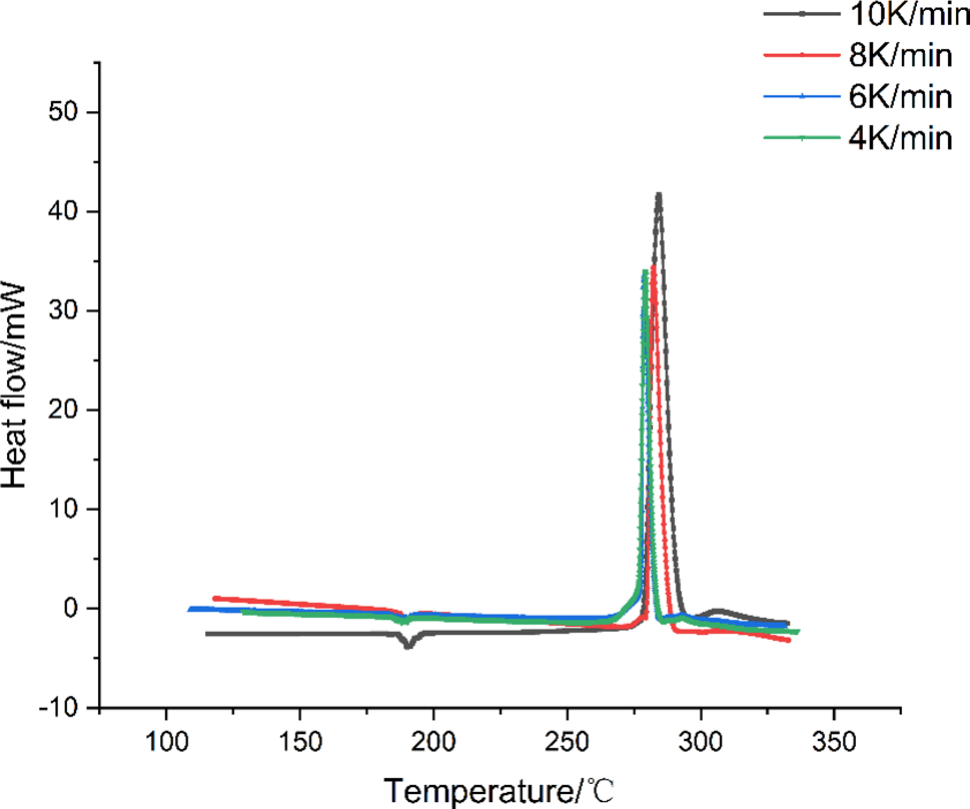
DSC curves of HMX at different heating rates under N_2_ atmosphere.

The DSC curves of HMX at various heating rates were obtained through DSC measurements conducted under dynamic test conditions, as depicted in Fig. 1. There is a distinct endothermal peak observed within the temperature range of 190.2 to 192.3 ℃. This peak can be attributed to the crystal transformation process from α-HMX to δ-HMX^24^. HMX is a well-known for crystallizing into four different forms. Among the various crystal forms, β-HMX stands out as the most stable form, exhibiting superior detonation performance and safety characteristics. α-HMX is a crystalline form that can be readily obtained through production and exhibits a high sensitivity. δ-HMX represents the crystal form prior to undergoing thermal decomposition. Each crystal form of HMX exhibits a distinct and stable range. When the temperature exceeds 188 °C, the α-HMX crystal undergoes a transformation process. The molar enthalpy range associated with the crystal transformation process is 3 ~ 9 kJ/mol^25–27^.

**Figure 2:**
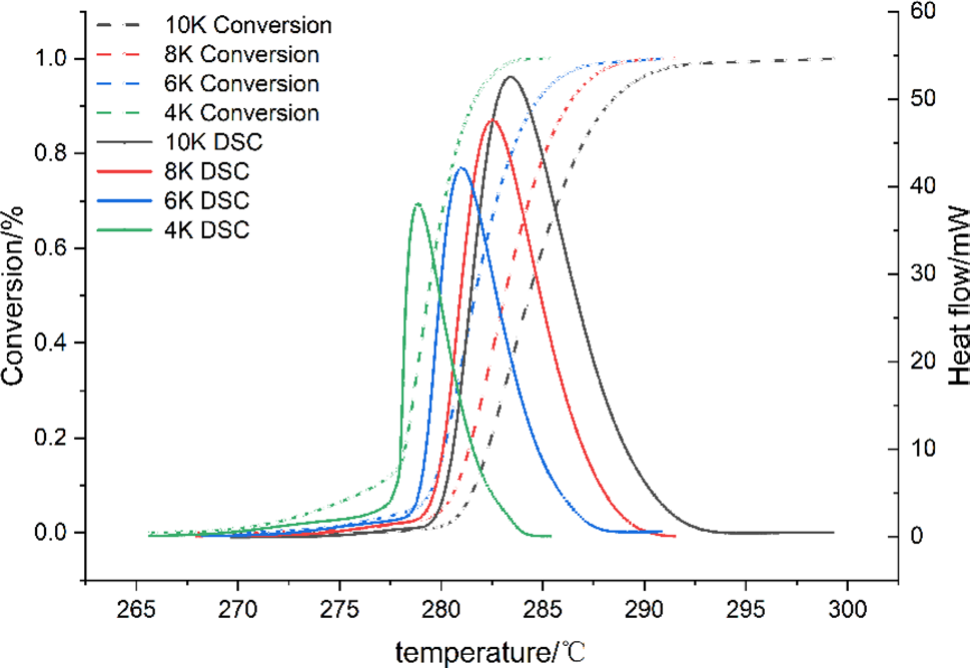
Decomposition conversion of HMX at different heating rates.

The Figure 2 shows the heat flow curves and the degree of HMX thermal decomposition can be determined by sequentially performing baseline reconstruction, temperature reconstruction, and deconvolution of the original data in TSS. The epitaxial onset temperature (T_0_), the inflection temperature (T_i_), the peak temperature (T_p_), and the decomposition termination temperature (T_f_) of HMX at different heating rates were listed in Table 1. The results of this study are basically consistent with those reported in the literature, confirming the reliability of the data.

**Table 1:** DSC data of HMX at different heating rates.

β (K/min)	T_0_(℃)	T_*i*_(℃)	T_*p*_(℃)	T_*f*_ (℃)	Literature
4	263.00	278.03	279.31	284.36	this study
6	263.00	279.24	279.48	284.68	this study
8	264.81	280.08	282.74	292.43	this study
10	265.77	280.72	284.19	294.94	this study
5	–	280.00	–	285.00	L. Patidar^28^
1	265.00	–	–	285.00	J. Kimura^5^
10	–	279.00	283.00	–	O. Ordzhonikidze^29^
20	–	285.00	290.00	–	G. Hussain ^12^

Taking the curve at a heating rate of 4 K/min as an example, it is evident that HMX starts to decompose at 275.4 °C, and the decomposition process is gradual. At the onset of decomposition, the exothermic rate of HMX decomposition increases rapidly and reaches its maxmium within 2.94 minutes. From the beginning to the end of the rapid decomposition process, the value of the heat conversion rate is increased from 13% to 100%. The duration of this process is 4.60 minutes, and the average increasing rate of the heat conversion rate is 18.91% per minute. This observation suggests that HMX should decompose rapidly and is difficult to control after experiencing the induction period of decomposition from T_0_ to T_i_ owing to the heat accumulation.

As the heating rate was elevated, the increase values of T_0_, T_i_, T_p_, and T_f_ were observed and it takes a longer time to reach the maximum decomposition rate. This observation suggests that the slow thermal decomposition initiates when the heating rate is low, and the accumulated heat accelerates the decomposition process.

### Kinetic parameter evaluation

**Figure 3:**
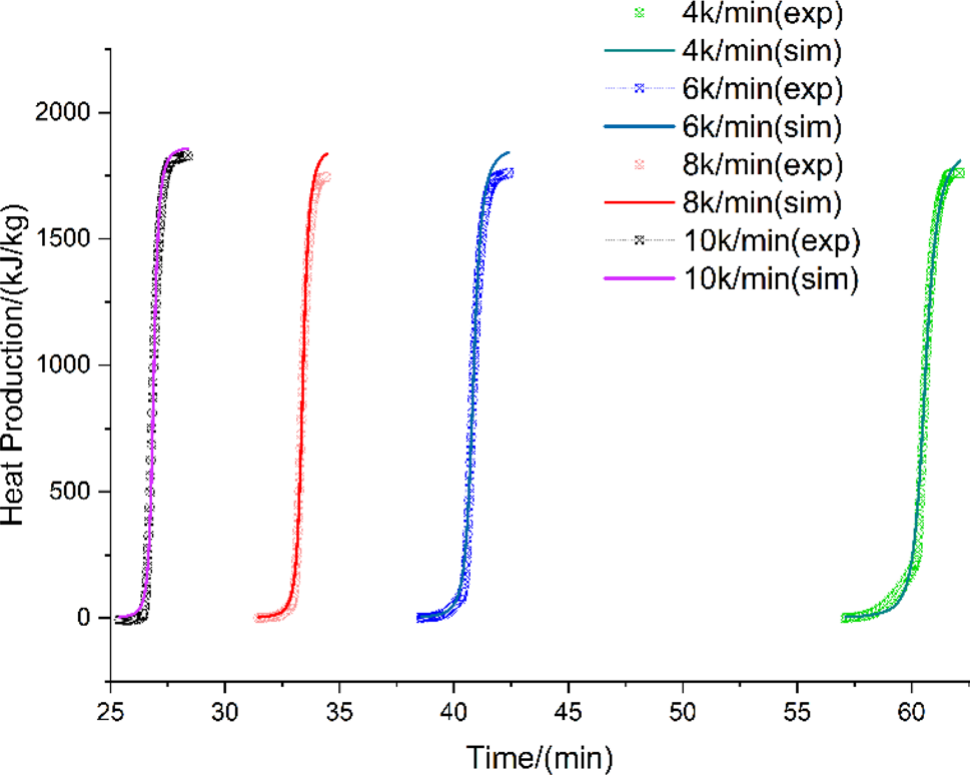
Experimental and simulated curves of heat production vs. time for HMX.

**Figure 4:**
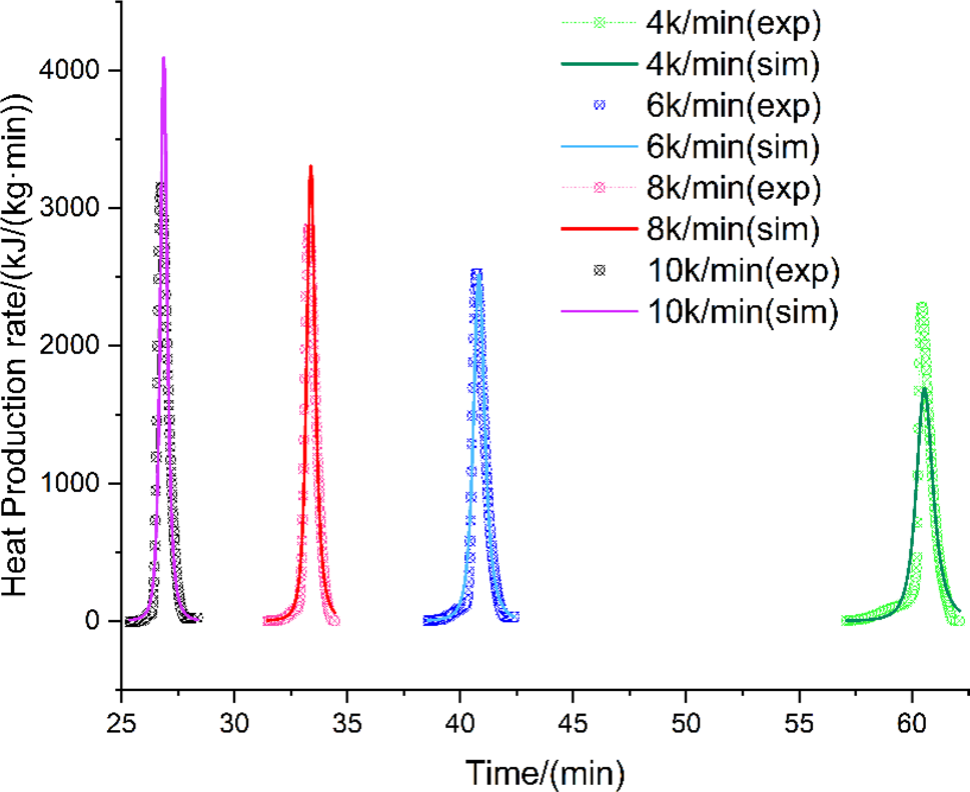
Experimental and simulated curves of heat production rate vs. time for HMX.

In order to assess the potential harm caused by the thermal decomposition of HMX, a kinetic model of thermal decomposition is required to describe the specific characteristics of the decomposition process. For the exothermic decomposition reaction of HMX, the autocatalytic reaction rate model was employed to accurately determine the kinetic parameters by fitting the experimental data. The heat production rate and heat production of HMX were simulated using TSS and compared with DSC experiments at the heating rates of 4, 6, 8, and 10 K/min. The heating rate used in the DSC test is also set between 4 and 10 K/min to obtain consistent and regular data. The results of the heat production and heat production rate are presented in Fig. 3 and Fig. 4, respectively. The simulation results exhibit strong agreement with the experimental findings, suggesting that the reaction model can effectively describe the thermal decomposition process of HMX. The correlation coefficients are presented in Table 2.

**Table 2:** Correlation coefficients for Figs. 3 and 4.

Correlation coefficient	4 K/min	6 K/min	8 K/min	10 K/min
Heat production	0.997	0.999	0.996	0.997
Heat production rate	0.945	0.977	0.965	0.924

Accordingly, the thermal decomposition kinetic model of HMX can be characterized as an autocatalytic reaction type. The apparent kinetic parameters of the reaction model, determined by TSS, are presented in Table 3. The comprehensive comparison results, obtained from the simulation of the autocatalytic model at different heating rates, are presented in Figs. 3 and 4. These figures serve to emphasize the dependability of the fitting outcomes.

**Table 3:** HMX thermal decomposition kinetic parameters derived from the TSS simulation.

Parameters	Units	Value
${\varvec{l}}{\varvec{n}}({\varvec{A}})$	$ln(1/s)$	93.81 ± 0.360
${\varvec{E}}$	kJ/mol	441.22 ± 1.680
${{\varvec{n}}}_{1}$	–	1.70 ± 0.014
${{\varvec{n}}}_{2}$	–	1.16 ± 0.014
${\varvec{l}}{\varvec{n}}({{\varvec{z}}}_{0})$	–	− 3.63 ± 0.030
${{\varvec{E}}}_{{\varvec{z}}}$	kJ/mol	9.85 ± 0.020
${\varvec{Q}}$	J/g	1861.51 ± 1.400

The obtained apparent activation energy of the thermal decomposition of HMX is 441.22 ± 1.68 kJ/mol, which closely aligns with the values of 432.2 kJ/mol^14^ and 428.49 ± 49 kJ/mol^29^ reported in the literature, which suggests that the obtained apparent activation energy is reliable. Therefore, it is resonable to use the calculated kinetic model and parameters to simulate the thermal explosion of HMX.

### Thermal explosion and runaway hazards analysis

The thermal decomposition mechanism is considered to be an intrinsic and inherent property of a substance, and the kinetic model and the associated parameters should not be influenced by the quality of the sample, the test mode, or choice of the test instrument. Although the kinetic model and its associated parameters derived from DSC experiments using a limited sample size may not be directly applicable to industrial production, it is still helpful to predict and simulate the risk of thermal explosion and runaway using this model, which has been confirmed by the consistence of the experiment and the simulation of the heat production and the heat production rate.

For complex multi-stage exothermic chemical reaction in a solid material, it is assumed that the process is not accompanied by pore-formation or phase transition. In this case, heat transfer in a solid is described by the thermal conductivity equation with nonlinear energy source. In the process of simulating thermal explosions, the heat transfer model can be represented by the following Eqs. ^19,30,31^:

Thermal conductivity equation:

4 $$\rho C_{P} \frac{\partial T}{{\partial t}} = div\left[ {\left( {\lambda (grad T} \right)} \right] + W$$

where $\rho$ is density; ${C}_{P}$ is the specific heat capacity; $\lambda$ is the thermal conductivity; $W$ is the thermal power; $T$ is the kelvin temperature; *div* is divergence; *grad* is gradient. By solving the heat equation, the temperature of an object with uneven temperature distribution changes over time and space can be obtained.

When time is zero, the initial conditions for temperature and transition are defined as ${T}_{0}$ and ${a}_{i0}$. In addition, several boundary conditions (BC) need to be defined individually on each surface of an object in order to solve the differential equation of heat conduction. The boundary conditions describe the thermal state at the boundary of the thermally conductive object and its interaction with the surrounding environment.

BC of the 1st kind:

5 $$\left. T \right|_{s} = T_{e} \left( T \right)$$

BC of the 2nd kind:

6 $$\left. q \right|_{s} = q\left( t \right)$$

BC of the 3rd kind:

7 $$- \lambda \left. {\frac{\partial T}{{\partial n}}} \right|_{s} = U\left( {T_{s} - T_{e} } \right)$$

BC of the 4th kind:

8 $$- \lambda \left. {\frac{\partial T}{{\partial n}}} \right|_{5} = \sigma \varepsilon_{eff} \left( {T_{s}^{4} - T{\text{e}}^{4} } \right)U\left( {T_{s} - T_{e} } \right)$$

where $q$ is the external specific heat flux; $n$ is the unit outer normal on the boundary; ${\varepsilon }_{eff}$ is the effective emissivity; $\sigma$ is the Stefan-Boltzmann constant. And The subscripts "s" and "e" denote the parameters pertaining to the boundary and the environment, respectively; *T* represents the kelvin temperature.

BC of the 1st kind assumes a high level of heat exchange between the system and the environment, disregarding the thermal resistance of the container wall. As a result, the system temperature is considered to be equal to the ambient temperature. BC of the 2nd kind signifies a heat flow gradient of zero, indicating the absence of heat transfer from a higher temperature region to a lower temperature region. This condition characterizes an adiabatic process, where no heat exchange occurs. BC of the 3rd kind pertains to the prevalent convective heat transfer process in biological systems. BC of the 4th pertains to the phenomenon of radiative heat transfer.

#### Simulation of thermal explosions

**Figure 5:**
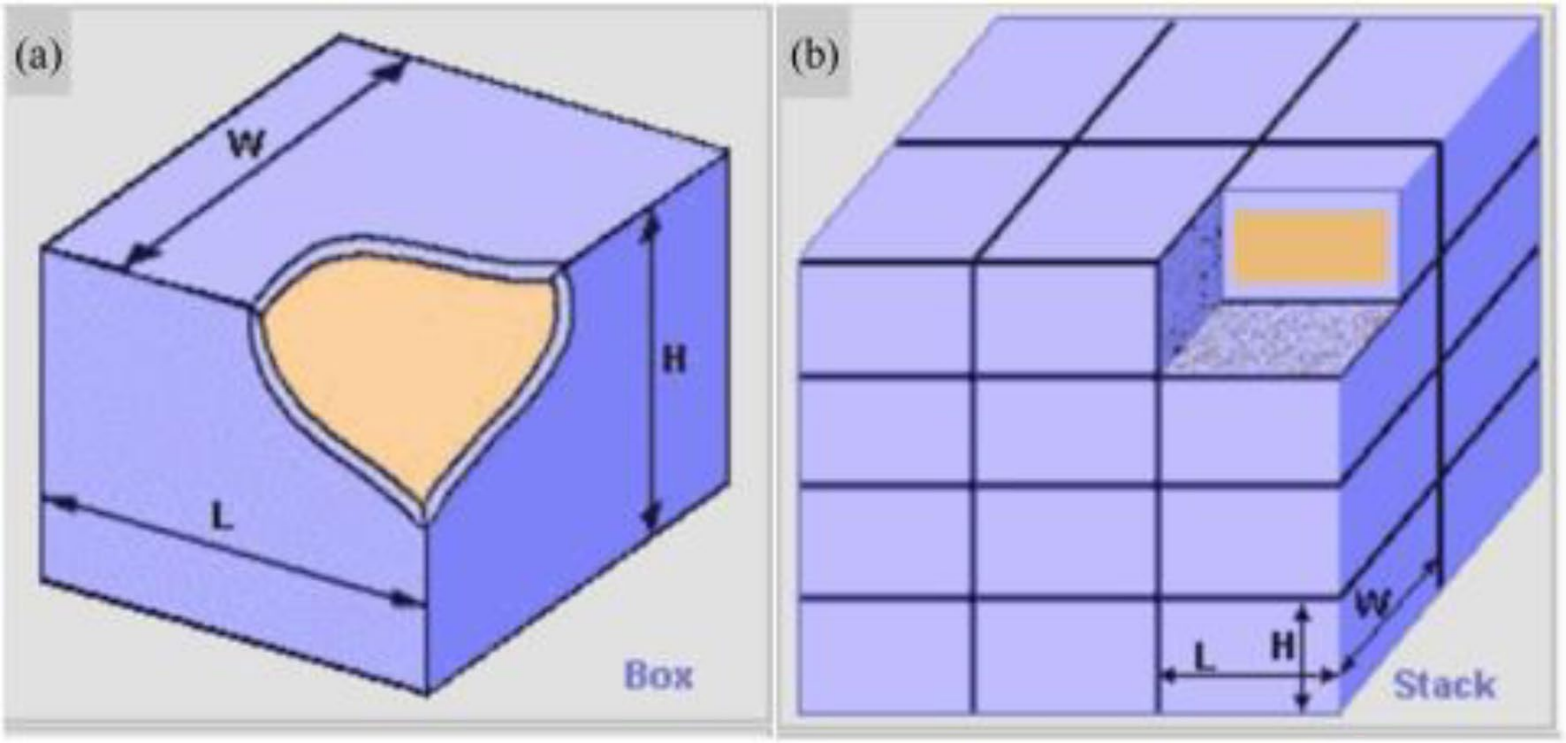
The geometry model of container^16^, (**a**) The standard 1 m^3^ container, (**b**) Stack boxes.

According to the United Nations' recommendations on the transportation of hazardous materials in large-scale operations, in order to streamline the process of delivery and loading, it is advised to utilize contemporary packaging methods for explosives. This involves the use of a standardized 1 m^3^ container specifically designed for explosives, or alternatively, employing multiple stacked packaging units^32,33^. HMX as a typical representative of explosives should also refer to this approach^17^. Therefore, the standard 1 m^3^ fiberboard container (L × W × H: 135 × 93 × 100 cm; shell thickness 4 cm) and the stack of 64 (27 × 27 × 27 cm; shell thickness 1 cm) boxes as the tank for storing about 1960 kg of HMX were used for the thermal explosion simulation to explore the thermal runaway consequence of HMX in storage or transportation process. The geometries of two packing methods^16^ are shown in Fig. 5, and the detailed thermo-physical parameters of sample and storage containers are listed in Table 4.

**Table 4:** The effective thermo-physical parameters.

Parameters	*c*_*p*_ (J/g/K)	${\varvec{\rho}}$(g/cm^3^)	${\varvec{\lambda}}$(W/m/K)
HMX	0.415 + 0.002 T	1.96	0.10
Fiberboard	1.700	0.75	0.30

Given the objective of examining the storage of HMX in air, the heat transfer model employed in this simulation is based on Newton's heat exchange law (7). The selection of this option is determined by the mode of heat transfer resulting from the disparity in temperature between the system and its surrounding environment. To determine the numerical solution of this equation, it was assumed that the initial temperature and transitions in each active region are uniformly distributed and all transitions are equal to zero. According to Newton's heat exchange law, the external boundary conditions are all set to: BC of the 3rd kind (ambient temperature, heat transfer coefficient 10 W/m^2^/K) and the internal interface is set to coupling. Considering the environments of different regions and seasons during storage and transportation, the ambient temperatures are set to 0 ℃, 25 ℃ and 40 ℃, respectively. Then, the thermal explosion behaviors of HMX under different packaging and environmental conditions have been simulated to determine the thermal explosion critical temperature (T_cr_) and induction period (*θ*).

**Figure 6:**
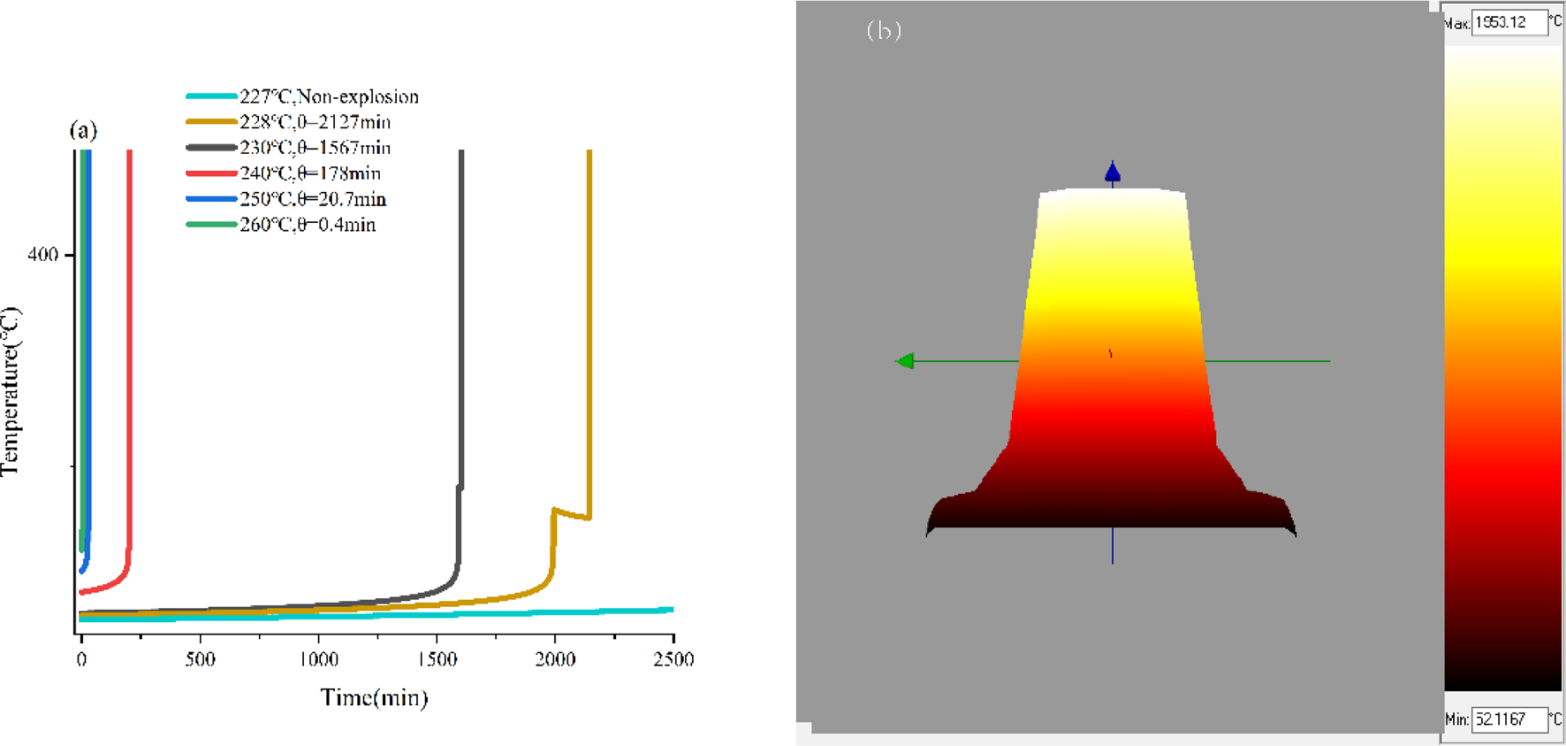
Simulated temperature vs. time of HMX under ambient temperature of 25 °C, (**a**) the standard 1 m^3^ container, (**b**) 3-D temperature distribution inside the material-side.

**Figure 7:**
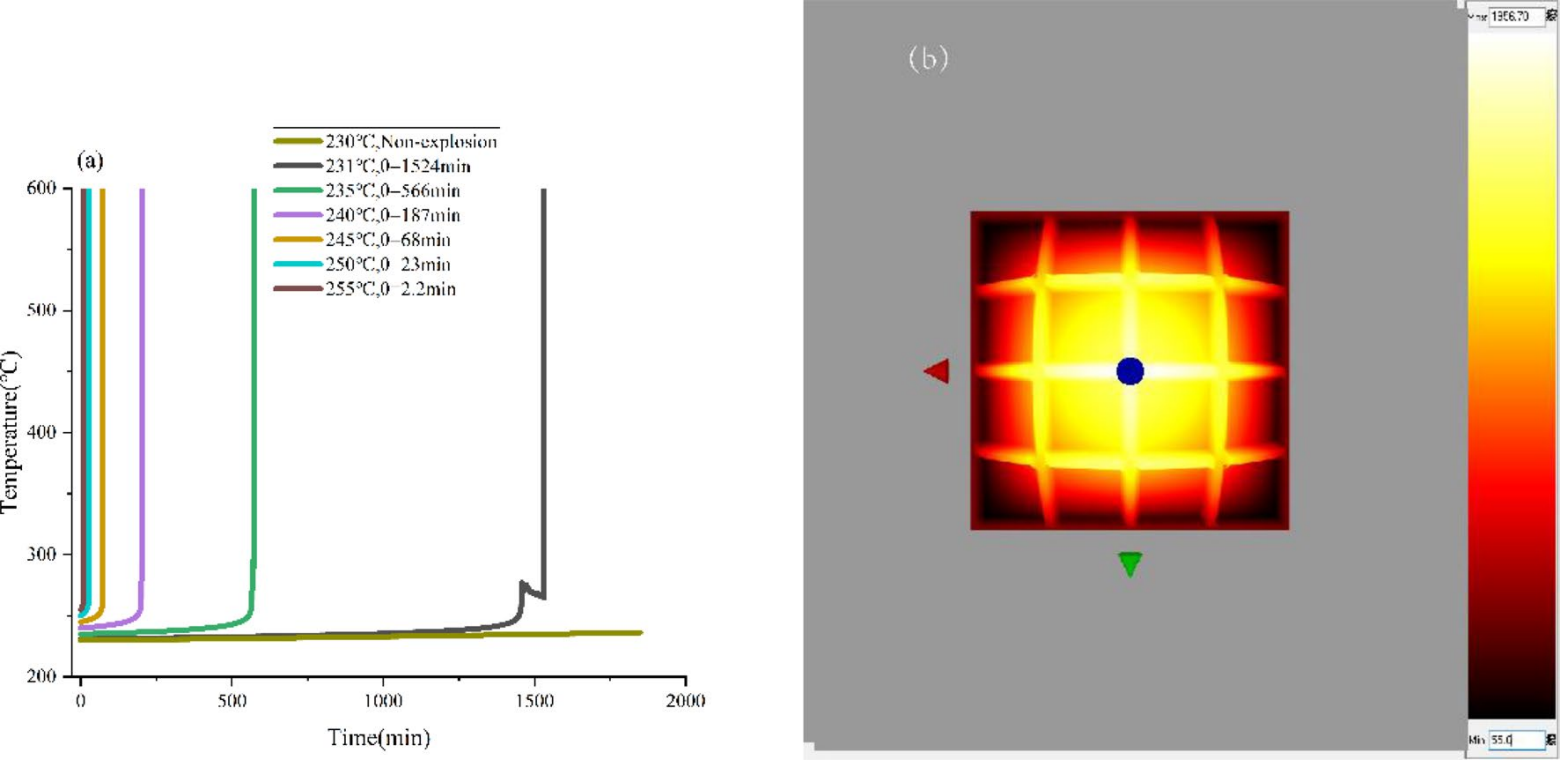
Simulated temperature vs. time of HMX under ambient temperature of 25 ℃, (**a**) the stack boxes, (**b**) 3-D temperature distribution inside the material-side.

The thermal explosion curves of the standard 1 m^3^ container and the stacked box at various initial system temperatures are shown in Fig. 6a and Fig. 7a, respectively. Assuming the ambient temperature was 25 ℃, the T_cr_ values of the standard 1 m^3^ container and the stacked box can be determined to be 227 ℃ and 230 ℃, respectively. Simulations of the thermal explosion behaviors of HMX were also performed at 0 ℃ and 40 ℃, respectively. The values of T_cr_ at the different ambient temperatures were listed in Table 4. In the simulation of thermal explosion, the complex mathematical calculation is used in the simulation. Although the scaling method of the simulation process is more universal, it remains an approximate approach. Consequently, it cannot completely ensure the accurate thermal equivalence of reaction systems with varying sizes and geometries.

The Fig. 6a and 7a demonstrate that when the heat release rate of thermal decomposition is below the cooling capacity of the surrounding environment, the system effectively conducts the generated heat to the environment, preventing heat accumulation within the system. When the system temperature exceeds the T_cr_, the decomposition process is accelerated. As a result, the heat generated exceeds the amount dissipated to the environment, leading to heat accumulation within the system. This further increases the rate of heat generation and ultimately results in a thermal explosion.

**Figure 8:**
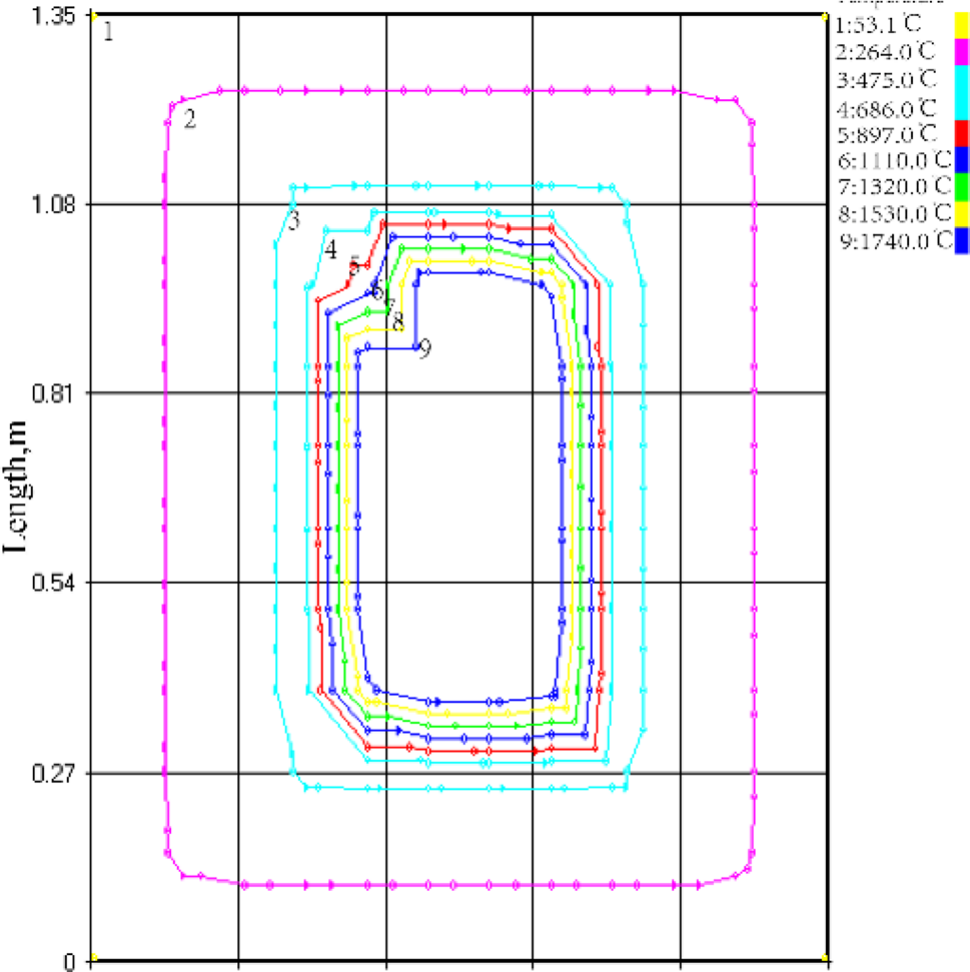
Simulated isotherm of HMX thermal explosion moment at ambient temperature of 25 °C and system temperature of 250 °C.

As shown in Fig. 6b, 7b and 8, the accumulated heat primarily leads to a significant temperature rise at the center of the entire system in the case of thermal runaway or thermal explosion. Such results can be attributed to the fact that the heat transfer rate at the system's edges is faster, and the heat can be moved out in time. while the heat transfer rate is slower at the center, leading to high heat accumulation.

According to the calculation results in Table 5, Fig. 6 and 7, the effects of the ambient temperature on the two packaging methods are similar. Compared with the method of storing in the stacking box, the shorter thermal explosion induction period at the same initial system temperature and the lower value of T_cr_ were observed when HMX was stored in the standard 1 m^3^ container. Therefore, storing HMX in the standard container of 1 m^3^ is more prone to thermal runaway, and the process may be more intense, which is associated with higher safety risks. When operating in large quantities, it is recommended to choose several shots (stacks) of packaging for transportation rather than storing all materials in one package for quick transportation and storage.

**Table 5:** The simulated T_cr_ under different ambient temperatures.

Ambient temperature (°C)	the standard 1 m^3^ container(°C)	the stack boxes(°C)
0	229	234
25	228	231
40	227	229

#### Simulation of the thermal explosion under different package conditions

**Figure 9:**
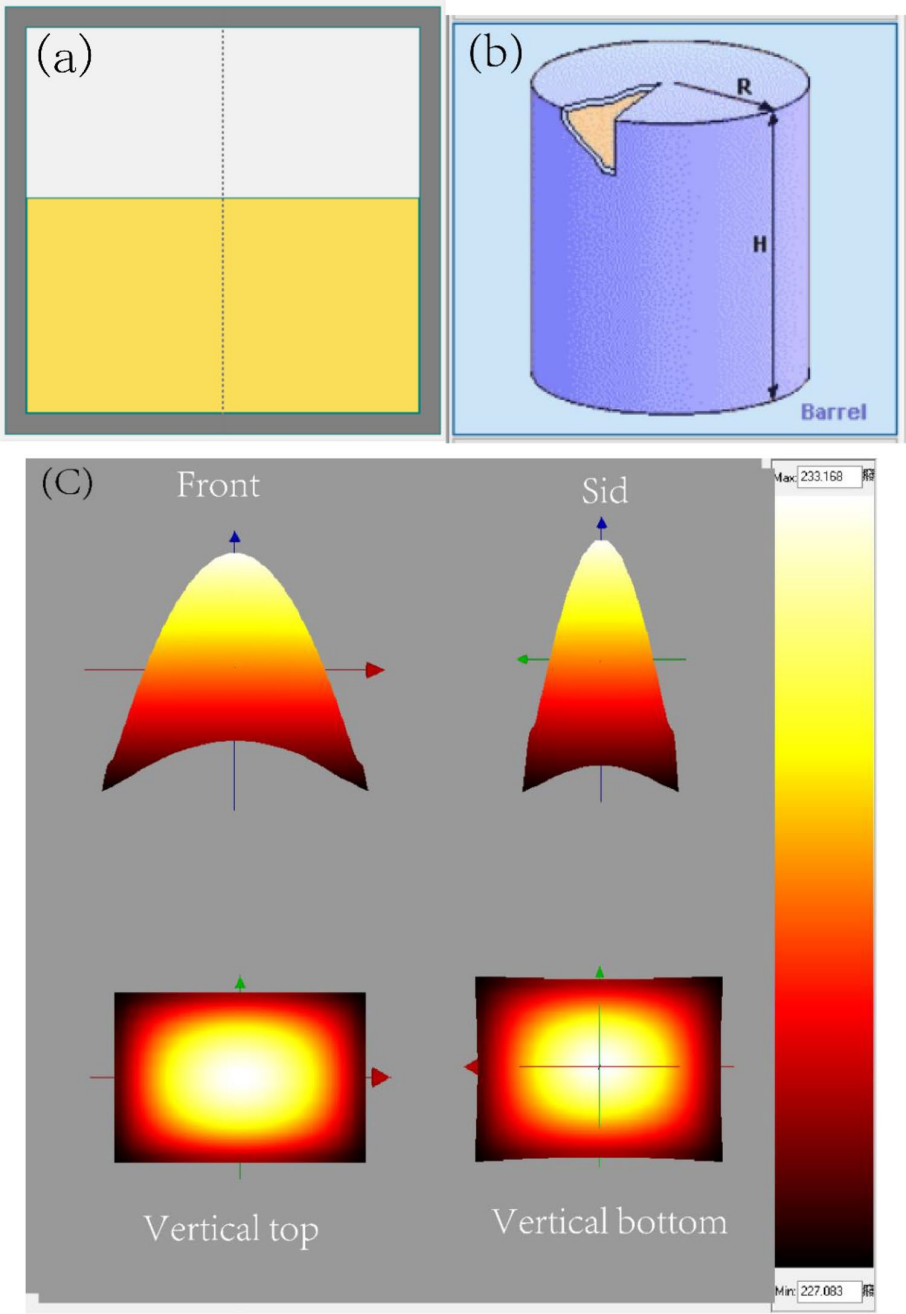
TSS simulation results of SADT, (**a**) The geometry of barrel, (**b**) HMX stored in the barrel^17^, (**c**) 3-D temperature distribution inside the material (20 kg, fiberboard barrel).

The assessment of thermal explosion hazard parameters can significantly contribute to optimize the transportation and storage conditions for chemicals. Additionally, it can provide valuable assistance in mitigating industrial risks. The parameters associated with thermal hazards encompass the self-accelerating decomposition temperature (SADT), control temperature (CT), and emergency temperature (ET)^34,35^, are obtained by the numerical calculations of the kinetic model while considering the influence of the geometry and boundary conditions of the container. TSS was used to simulate the storage of 20 kg of HMX in fiberboard barrels, as shown in Fig. 9a,b, and the detailed thermo-physical parameters of sample and the storage containers are listed in Table 3. The 3D temperature distribution result in Fig. 9c suggests that a overheat of 6.08 °C in the center is observed when the ambient temperature is 227 °C in a 20 kg of commercial package.

The prediction of the relevant dimensional parameters and boundary conditions for the packages with different amounts and the obtained thermal explosion parameters at 25 °C are listed inf Table 6.

**Table 6:** Thermal hazard boundary parameters of HMX.

Mass of sample (kg)	Size (R × H)(m)	U(W/m^2^/K)	Void fraction (%)	SADT (℃)	CT (℃)	ET (℃)
5	0.10 × 0.20	6.5	44.0	228	218	223
25	0.15 × 0.30	5.8	41.1	227	217	222
50	0.20 × 0.40	5.4	40.0	226	216	221

As the mass of HMX in the package increases, the SADT gradually decreases, indicating that when more materials are piled up together, the thermal runaway occurs more easily. Under typical conditions, numerical simulation serves as a reliable approach for accurately determining the SADT once the specific packaging specifications have been established. The manuscript employs standardized containers and packaging that are specifically designed for explosives, adhering to the prescribed packaging specifications. The data obtained holds significant reference value for manufacturers in practical applications.

## Limitations

The rule of change in SADT and other data derived from simulation can be applied to real-world scenarios. However, it should be noted that the specific data values may be influenced by factors of product purity, environmental humidity, and temperature distribution.

## Conclusions

By conducting non-isothermal DSC measurements on HMX at different heating rates, the kinetic parameters that govern the decomposition of HMX were determined. Subsequently, a decomposition kinetic model was formulated, and the essential thermal hazard parameters were predicted. After conducting the analysis, a number of conclusions can be deduced.

A. The non-isothermal DSC results show that the thermal decomposition of HMX firstly undergoes crystal transformation; once the decomposition temperature was reached, the decomposition of HMX proceeds at a very fast rate and is completed rapidly. The kinetic model of thermal decomposition was determined to be autocatalytic by using kinetic simulation for the exothermic part of the curve. The obtained apparent activation energy of the thermal decomposition of HMX is 441.22 ± 1.68 kJ/mol, the pre-exponential factor satisfies ln(A) = 93.81 ± 0.360.
B. In order to address the issue of the data obtained from the DSC test being unsuitable for direct application in practical scenarios, the thermal runaway and explosion of HMX were simulated, and thermal hazard parameters such as T_cr_ and SADT were predicted based on the kinetic model of thermal decomposition. According to the prediction, it can be found that several rounds of packaging (stacking) is a safer method than the concentrated stacking during the transportation and storage of large volume and mass of HMX. Therefore, it is recommended to choose several shots (stacks) of packaging for transportation rather than storing all materials in one package for quick transportation and storage, and the mass of HMX in packaging materials should be carefully controlled, as an excessive amount can lead to a decrease in the SADT then an increased risk of thermal runaway. Due to the hazardous nature of HMX as an explosive material, the occurrence of thermal runaway can lead to catastrophic consequences.
C. Through simulation, it is found that when HMX reaches the decomposition temperature, the system temperature will reach the maximum temperature at a very fast speed during the autocatalytic decomposition. And the center of the system usually shows the highest temperature. Therefore, even if the ambient temperature is low, the temperature of the center point still rises rapidly and reach T_cr_ easily, resulting into the thermal runaway and explosion. Therefore, under certain packaging and storage conditions, the study on how to control the temperature of the center point remains to be completed.

## Data Availability

The datasets used and/or analysed during the current study available from the corresponding author on reasonable request.
